# Automatic classification of fine-scale mountain vegetation based on mountain altitudinal belt

**DOI:** 10.1371/journal.pone.0238165

**Published:** 2020-08-25

**Authors:** Junyao Zhang, Yonghui Yao, Nandongzhu Suo

**Affiliations:** 1 Skate Key Laboratory of Resources and Environmental Information System, Institute of Geographic Sciences and Natural Resources Research, Chinese Academy of Science, Beijing, China; 2 University of Chinese Academy of Sciences, Beijing, China; Trent University, CANADA

## Abstract

Vegetation mapping is of considerable significance to both geoscience and mountain ecology, and the improved resolution of remote sensing images makes it possible to map vegetation at a finer scale. While the automatic classification of vegetation has gradually become a research hotspot, real-time and rapid collection of samples has become a bottleneck. How to achieve fine-scale classification and automatic sample selection at the same time needs further study. Stratified sampling based on appropriate prior knowledge is an effective sampling method for geospatial objects. Therefore, based on the idea of stratified sampling, this paper used the following three steps to realize the automatic selection of representative samples and classification of fine-scale mountain vegetation: 1) using Mountain Altitudinal Belt (MAB) distribution information to stratify the study area into multiple vegetation belts; 2) selecting and correcting samples through iterative clustering at each belt automatically; 3) using RF (Random Forest) classifier with strong robustness to achieve automatic classification. The average sample accuracy of nine vegetation formations was 0.933, and the total accuracy of the classification result was 92.2%, with the kappa coefficient of 0.910. The results showed that this method could automatically select high-quality samples and obtain a high-accuracy vegetation map. Compared with the traditional vegetation mapping method, this method greatly improved the efficiency, which is of great significance for the fine-scale mountain vegetation mapping in large-scale areas.

## Introduction

As an essential component of mountain ecosystems, vegetation is the basis of mountain ecological services and an indicator that responds to environmental change [1]. Therefore, vegetation mapping is of considerable significance to both geoscience and mountain ecology [2,3]. In recent years, with the development of aerospace technology, remote sensing has become a conventional means of vegetation mapping. With the improvement of image resolution, it is possible to map vegetation at a finer scale. Meanwhile, the automatic classification of vegetation has gradually become a research hotspot [4–7]. Traditional automatic classification of fine-scale vegetation was mainly based on feature selection and improvement of the classifier, and the samples used in the process were mostly selected manually [8–10]. Its automation was only an improvement over the degree of visual interpretation, and the real-time and rapid collection of samples has become a bottleneck of automatic classification [11–14].

With the increasing requirement of efficiency, some automatic sampling methods were proposed. A method of using prior maps is widely used. For example, Jin *et al*. [15] developed an approach named Alaska Land Cover Update 2011-Vegetation (AKUP11-VEG) to update land cover in vegetation disturbed and successional areas from 2001 to 2011, and used the initial land cover map of 2011 as the training dataset for decision tree classification; Mellor *et al*. [16] derived training data from 766 2×2 km digital aerial photograph interpreted (API) land cover maps for RF (Random Forest) classifier. However, due to label errors or classification errors in the prior maps, samples selected on this basis are often affected by error propagation [17]. To solve this problem, researchers have proposed some methods to filter samples that may contain errors. One way is filtering by using spatial information. Thus, Jiang *et al*. [18] discarded the samples in the joint region of different land cover with spatial buffer analysis to prevent the influence of land cover changes. Zhang *et al*. [11] extracted samples from the land cover product with classification confidence >50%, and only retained the pixel locations with the same land cover class in the surrounding eight pixels. The other way is filtering by using attribute information. Thus, Waldner *et al*. [19] extracted samples from the baseline land cover map and iteratively trimmed samples to identify statistical outliers. Matton *et al*. [20] proposed an automated method for annual cropland mapping and cleaned samples by iterative trimming with a threshold α of 0.01. Although the method using prior maps greatly improves the automation level of sample selection, the following problems persist: 1) errors due to changes that have occurred since the production date of prior maps, 2) errors due to different spatial resolution between the datasets or geo-locations [21], and 3) the classification systems of prior maps may restrict the fitness of new research. All these problems restrict the accuracy of the selected samples. More importantly, the prior maps used by this method are interpretation results at an earlier state in the same study area, which means that this method is more suitable for map updating such as change detection than for un-interpreted areas. Other methods mostly select samples by obtaining prior knowledge of sample population and setting threshold. However, the samples obtained by these methods usually correspond to vegetation/non-vegetation or coniferous forest/broad-leaved forest, which is not accurate enough for fine-scale vegetation classification [22–24]. Therefore, how to realize the automatic sample selection, which is a key problem of automatic classification of fine-scale vegetation, remains to be further studied.

The research showed that the selection of samples had a significant effect on the classification results [25]. Therefore, it is necessary to ensure sample accuracy while selecting samples in an automatic way [21,26,27]. For geospatial objects with spatial autocorrelation, classical sampling methods such as random sampling and systematic sampling assume that samples are entirely independent of each other, which leads to an underestimate of sampling error. Compared with the classical sampling methods, stratified sampling has the following advantages [28]: 1) the distribution of samples is more dispersed and uniform, which reduces the possibility of sample information overlapping, thus reducing the information loss; 2) the sample variance is equal to the interlayer variance instead of the population variance, which reduces the uncertainty of samples. It has been proved that stratified sampling with appropriate prior knowledge can bring better sample accuracy [26,29–32].

For vegetation classification, the close dependence of plant growth on hydrothermal conditions is an important prior knowledge. Mountain vegetation presents regular zonal arrangements characteristics with the increase of altitude, which is called the mountain altitudinal belt (MAB) [33,34]. The division of MAB is based on a response model that treats types of vegetation as a product of topography and climate. Therefore, MAB represents a relatively stable geographical regional differentiation phenomenon and reflects the spatial-temporal relationship between vegetation distribution and environmental factors [35,36]. Based on MAB, the study area can be divided into several vegetation belts, and the corresponding type of vegetation of each vegetation belt is the main species growing in this area. Compared with selecting samples in the whole study area, selecting samples in each belt can effectively reduce the interference of other types of vegetation, and the samples selected by this method are more representative. Moreover, MAB distribution information is generally summarized by researchers in the process of mountain surveys, and it can also be obtained from most mountain investigation reports or vegetation survey data, so it is a convenient and reliable prior knowledge for mountain vegetation classification [37–39].

The objective of this paper is to realize automatic sample selection and fine-scale mountain vegetation classification. The “fine-scale vegetation” implies that the minimum classification unit in this study is vegetation formation, rather than vegetation type group or vegetation type. In the classification system of vegetation, vegetation type group is the highest classification unit, which is mainly divided according to the morphological characteristics of the constructive species communities but also contains certain ecological content. Vegetation type and sub-type are higher classification units between vegetation type group and vegetation formation. The vegetation type is composed of the constructive species with the same or similar life type and the plant communities with consistent ecological relationship and hydrothermal conditions. Vegetation sub-type is an auxiliary unit of vegetation type. It reflects the difference in climate subzone or the differences in vegetation lamellar structure caused by the differences of certain landform and matrix conditions. Vegetation formation has the same or similar constructive species or co-constructed species, and it is the basic mapping unit of vegetation maps [40].

The research area is Taibai Mountain in the north-south transition zone (Qinling Mountains) of China, which has vegetation with obvious altitudinal distribution and sufficient survey data. Compilation of fine-scale vegetation maps in the transitional zone is essential for an in-depth study of the spatial-temporal variation rules of vegetation. It has great significance in revealing geo-ecological patterns [40]. However, the difficulty of vegetation classification is increased by the absence of distinct boundaries between vegetation formations. Therefore, mapping the vegetation distribution with multi-resolution, multi-source, and multi-phase images will be more accurate. The images used for mapping in this study were mainly ZY-3 (Resources satellite three) satellite images with a resolution of 2m, GF-1 (Gaofen-1) satellite images with a resolution of 16 m and GF-2 (Gaofen-2) satellite images with a resolution of 0.8 m.

Since the unit of MAB is hectometer, there may be problems of insufficient precision and inaccurate definition of the junction of vegetation belts. Addressing these problems, in each belt, we clustered and selected the optimal samples rather than taking all objects as the samples, to eliminate the possibility of mixing other vegetation formation samples in the current vegetation formation samples.

For a remote sensing mapping task, samples are the prerequisite for faster and more accurate classification, but the ultimate goal is to obtain a map with high accuracy. Therefore, we compared two commonly used classifiers to obtain a more accurate vegetation map. One is RF classifier, which is widely used because of its strong robustness to outliers and its faster calculation speed [41], and the other is KNN (K-NearestNeighbor) classifier with a simple algorithm and high accuracy [42,43]. The main process of mapping in this study included the following steps: 1) multi-scale image segmentation in eCognition; 2) using MAB distribution information as prior knowledge to construct terrain constraint factors which play a constraint role on research objects by using appropriate terrain factors; 3) using terrain constraint factors to divide the study area into 8 vegetation belts and selecting samples by iterative clustering within each vegetation belt based on the idea of stratified sampling; 4) using a more accurate classifier (RF or KNN) to realize fine-scale vegetation classification. Based on the results, we discussed the vegetation growth distribution law reflected in the classification results and analyzed the accuracy of the samples from three perspectives (north\south slopes, west\middle\east regions and overall accuracy of the entire Taibai Mountain) to guide future work.

## Materials and methods

### Study area

**Fig 1:**
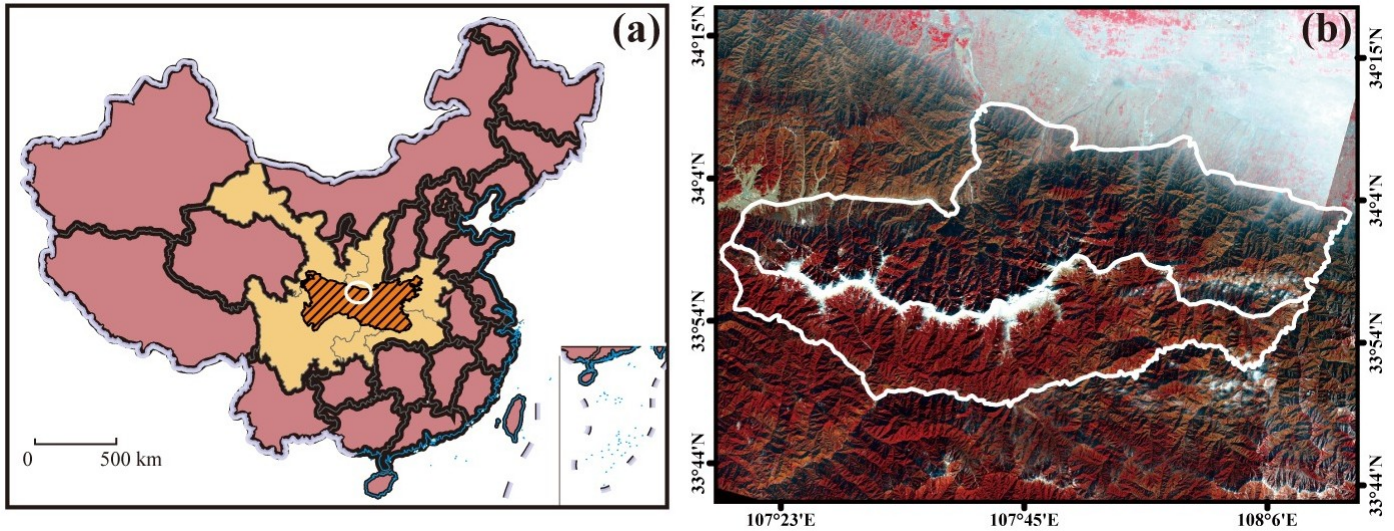
Extent of the study area. **(a)** The location of Qinling Mountains in China. The basemap was downloaded from http://bzdt.ch.mnr.gov.cn/index.html, and its figure number is GS(2019)1675. **(b)** Taibai Mountain, Landsat 8 image with a resolution of 15m, false color image (near-infrared (NIR), Red, Green), February 2017). The image is for illustrative purposes only.

Taibai Mountain (33°40'- 34°10'N and 107°19'- 107°58'E) is the highest mountain in Eastern China and located in Shaanxi Province with an altitude of 3771.2 m. It is also the main peak of the Qinling Mountains, a climate boundary between the warm temperate and the subtropical zones in Eastern China. The total area of Taibai Mountain is approximately 2,113.24 km^2^. Fig 1 shows the location of Taibai Mountain in the Qinling Mountains and China.

Under the influence of mountain height difference and atmospheric circulation, the climates of the north and south slopes of Taibai Mountain are different, with typical subalpine climate characteristics. From the perspective of altitudinal climate differences, a warm temperate zone, a temperate zone, a cold temperate zone, a cold zone, and an alpine cold zone are successively distributed from the foot to the top of the mountain [44]. Correspondingly, environmental and biological factors such as landform, soil, and vegetation also present altitudinal patterns [45]. The vegetation formations on the north and south slopes of Taibai Mountain are the same, but the altitudinal distribution ranges of the same vegetation formations are significantly different [46]. From the foot to the top of Taibai Mountain, a deciduous oak forest belt, a birch forest belt, a coniferous forest belt, and an alpine shrub meadow belt are successively distributed, and sub-belts are formed in each belt due to the variation and interaction of biological and non-biological factors [47].

### Study data

#### MAB distribution information of Taibai Mountain

**Fig 2:**
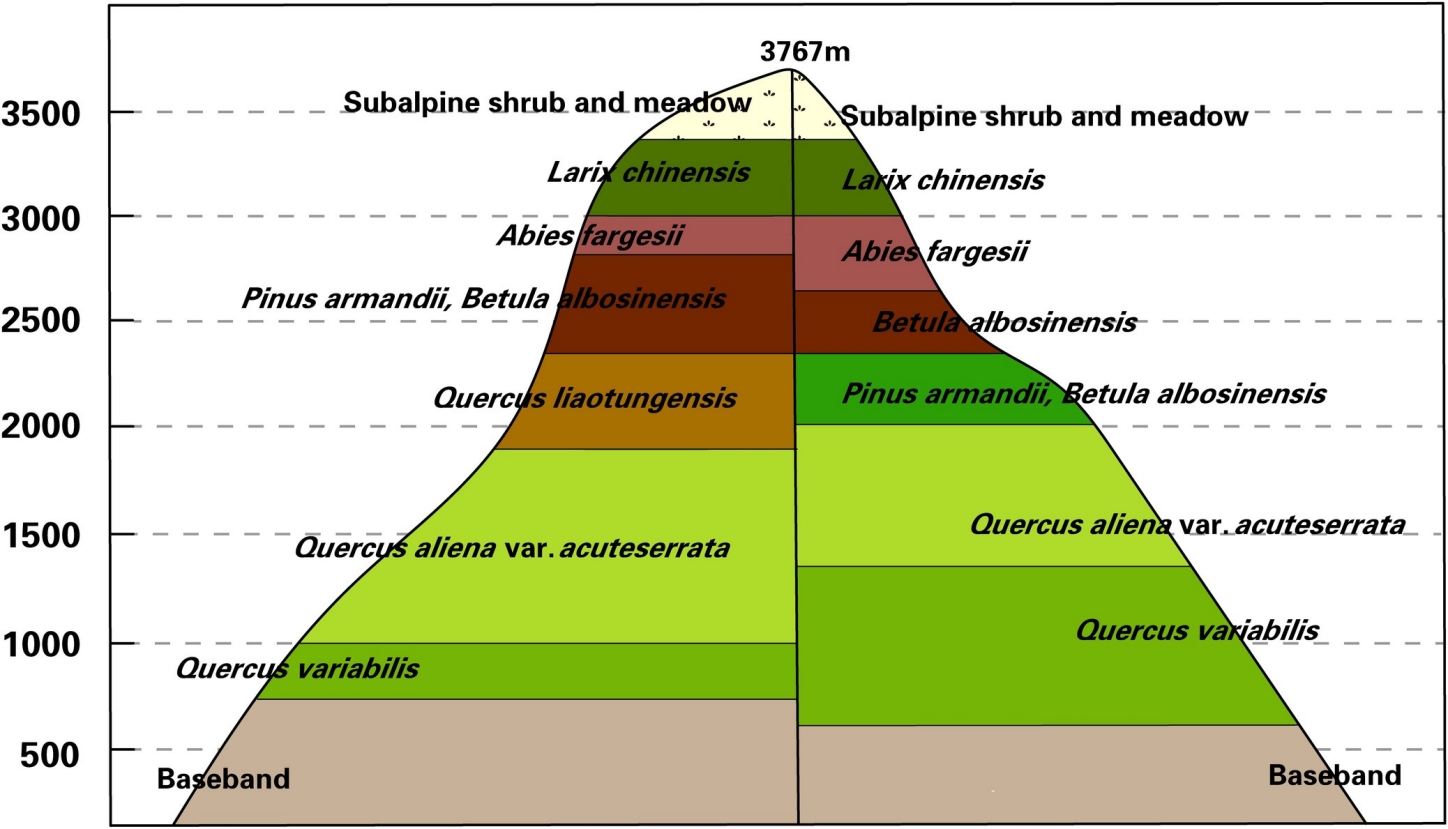
MAB distribution information of north and south slopes of the Taibai Mountain. The MAB distribution information was referenced to Fang & Gao [48] and Li [49].

The MAB distribution information, which was collated by Fang & Gao [48] and Li [49], was investigated and verified in a field survey of Taibai Mountain in June 2018. We can obtain the distribution ranges of vegetation formations and their spatial adjacency from the MAB distribution information. The vegetation formations on the north and south slopes were roughly the same, but the altitude distribution ranges of the vegetation formations were significantly different, as shown in Fig 2. On the north slope, the following vegetation formations are distributed from the foot to the top of Taibai Mountain: Basal zone (0–800 m), *Quercus variabilis* forest (800–1000 m), *Quercus aliena* var. *acuteserrata* forest (1000–1900 m), *Quercus liaotungensis* forest (1900–2300 m), mixed forests of *Betula albosinensis* with *Pinus armandii* (2300–2700 m), *Betula albosinensis* var. *septentrionalis* forest (2700–2800 m), *Abies fargesii* forest (2800–3000 m), *Larix chinensis* forest (3000–3400 m), and subalpine shrub and meadow (3400–3777 m). On the south slope, the following vegetation formations are distributed from the foot to the top of Taibai Mountain: Basal zone (0–750 m), *Quercus variabilis* forest (750–1300 m), *Quercus aliena* var. *acuteserrata* forest (1300–2000 m), mixed forests of *Betula albosinensis* with *Pinus armandii* (2000–2300 m), mixed forests of *Betula albosinensis* with *Betula albosinensis* var. *septentrionalis* (2300–2650 m), *Abies fargesii* forest (2650–3000 m), *Larix chinensis* forest (3000–3400 m), and subalpine shrub and meadow (3400–3777 m). In addition, the *Quercus liaotungensis* only grows in the north slope.

The MAB distribution information was primarily used as prior knowledge to stratify the study area and assist the automatic sample selection.

#### Remote sensing data

The remote sensing data used in this study were ZY-3 satellite images with a resolution of 2 m, GF-1 satellite images with a resolution of 16 m and GF-2 satellite images with a resolution of 0.8 m, as shown in Table 1. All these images were purchased from “Image Sky”, which is a company under “Geo-Science and Technology Service Network, CAS”. The multi-temporal GF-1 images with a resolution of 16 m were used for determining the approximate distribution range of different vegetation formations, GF-2 images with a resolution of 0.8 m were used for further verifying the accuracy of samples and classification, and ZY-3 images with a resolution of 2 m were used for segmentation and classification in this study. Since the images were preprocessed by radiometric correction and image fusion (the fusion of panchromatic (PAN) image and multispectral image) in ENVI (The Environment for Visualizing Images) v5.2 software, we clipped the images according to the vector boundary of the study area after a geometric correction. Besides, the 1:10,000 DSM (Digital Surface Model) data (resolution 10 m) generated from the ZY-3 images were used for building the terrain constraint factors.

**Table 1:** Remote sensing satellite image data information.

Satellite	Bands	Spatial Resolution (m)	Radiation resolution (bit)	Observation Date
GF-2	PAN+RGB+NIR	0.8	10	September 2017
ZY-3	PAN+RGB+NIR	2	10	January 2017
GF-1	PAN+RGB+NIR	16	10	January 2017/ September 2017

#### Validation data

The validation data used in this study included field sampling point data and a 1:50,000 visual interpretation vegetation map of Taibai Mountain compiled by Yao *et al*. [40]. The 1:50,000 visual interpretation results were primarily used to verify the accuracy of the vegetation classification results, and the field sampling point data were used to verify the accuracy of the selected samples. Field sampling point data were gathered from the field survey of Taibai Mountain from June 8th-15th, 2018, with a total of 86 points, and included the geographical location, vegetation type group, vegetation type/subtype, vegetation formation, and other attribute information.

## Methods

**Fig 3:**
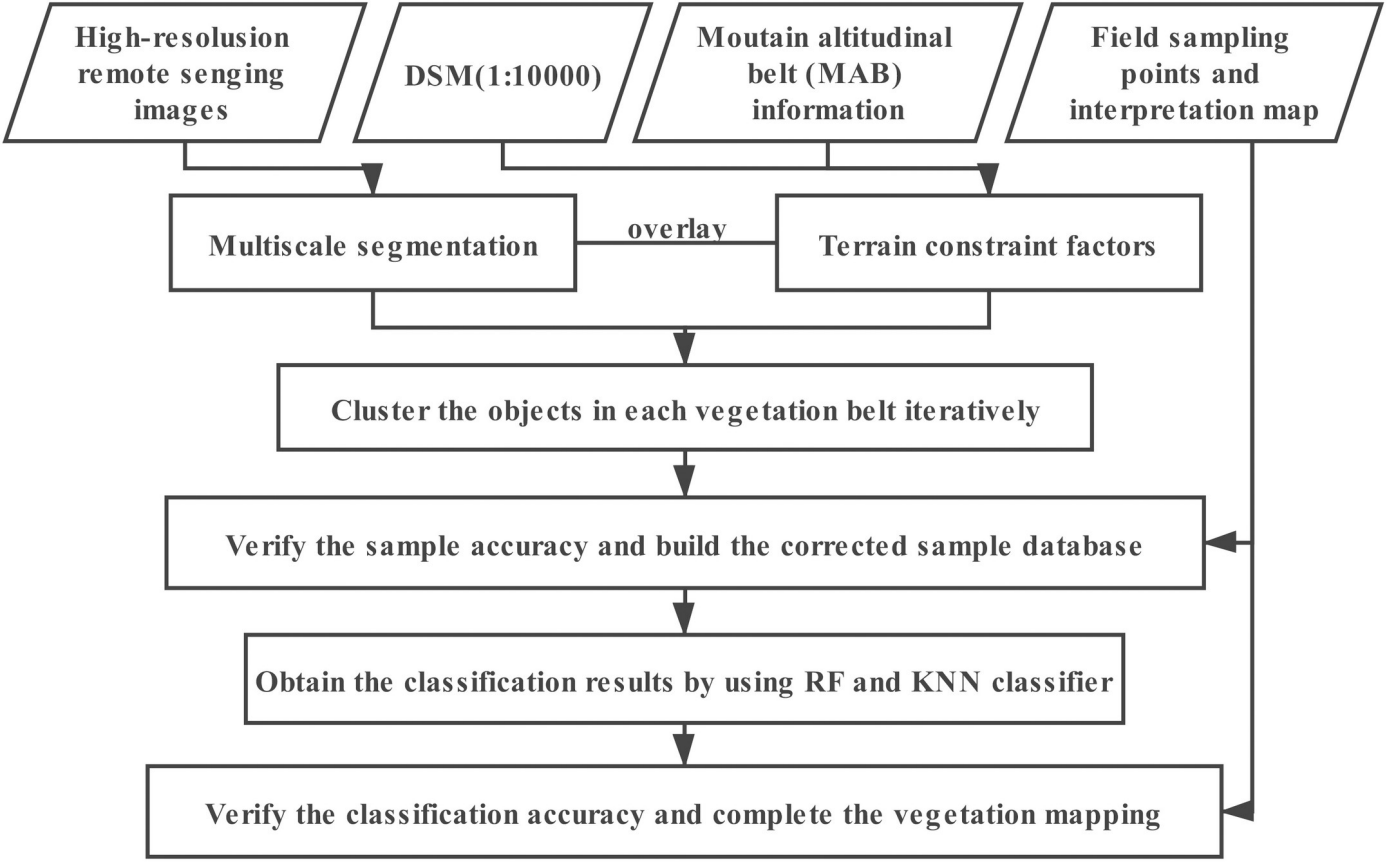
The main steps of this study. Flowchart showing the major steps involved in the vegetation mapping process.

A workflow of mapping process is shown in Fig 3.

### Multi-scale image segmentation

This study used the multi-scale segmentation algorithm in eCognition v8.9 software (Definiens Imaging, Germany), which is a widely used segmentation method based on regional growth and merging. The procedure for the multi-scale image segmentation starts with each pixel forming one image object or region. At each step, a pair of image objects are merged into one larger object. The merging decision is based on local homogeneity criteria, describing the similarity of adjacent image objects [50,51].

The segmentation process used auxiliary information including DSM data and texture feature for the following reasons: 1) using DSM data in segmentation can effectively refine the image where it was affected by mountain shadows; 2) using texture features in segmentation can effectively merge homogeneous objects to prevent an excessive number of objects [52]. Mean-variance was chosen as the judging standard for segmentation scale. The principle is that the purer the objects in the image layer, the higher the spectral difference between the objects and neighbors, which means the higher the mean-variance [53]. By drawing a broken line graph of the mean-variance and segmentation scale from 100 to 500 (see S1 Fig for a broken line graph), the peak value in the graph was the corresponding segmentation scale. The segmentation process was divided into two layers. The segmentation scale of Layer 1 was 360 and was used to extract non-vegetation areas, such as buildings and roads. The segmentation scale of Layer 2 was 140 and was used to select samples and extract specific vegetation group information (see S2 Fig for the segmentation results with the scale of 360 and 140). Additionally, six bands were utilized in the segmentation in this study. Due to the high reflective characteristics of vegetation in the NIR band, it was found that ideal segmentation results can be obtained when the weight of NIR band was larger than that of other bands, and there was no obvious change between the segmentation results of other bands with different weights (see S3 Fig for segmentation results under different band weight combinations). Therefore, the weight of NIR was set at 2, and the weight of other bands was set at 1. Moreover, the shape factor and compactness factor were selected after repeated experiments, as displayed in Table 2. Finally, objects with the spectrum, texture, terrain, and other information were obtained for subsequent classification after image segmentation.

**Table 2:** Parameters for image segmentation.

Layers	Extracted information	Segmentation scale	Shape factor	Compactness factor
Layer1	Non-vegetation area, including buildings, roads, etc.	360	0.2	0.6
Layer2	*Quercus variabilis* forest, *Quercus aliena* var. *acuteserrata* forest, *Quercus liaotungensis* forest, *Pinus armandii* forest, Birch forest, *Abies fargesii* forest, *Larix chinensis* forest, Subalpine shrub and meadow, Mixed conifer and broadleaved forest, Cultivated plants	140	0.2	0.6

### Automatic sample selection based on MAB distribution information

Based on the idea of stratified sampling, we used MAB as prior knowledge to construct terrain constraint factors for stratification, and selected samples automatically by iterative clustering in each vegetation belt. The specific methods were as follows.

#### (1) Constructing terrain constraint factors based on MAB distribution information

**Fig 4:**
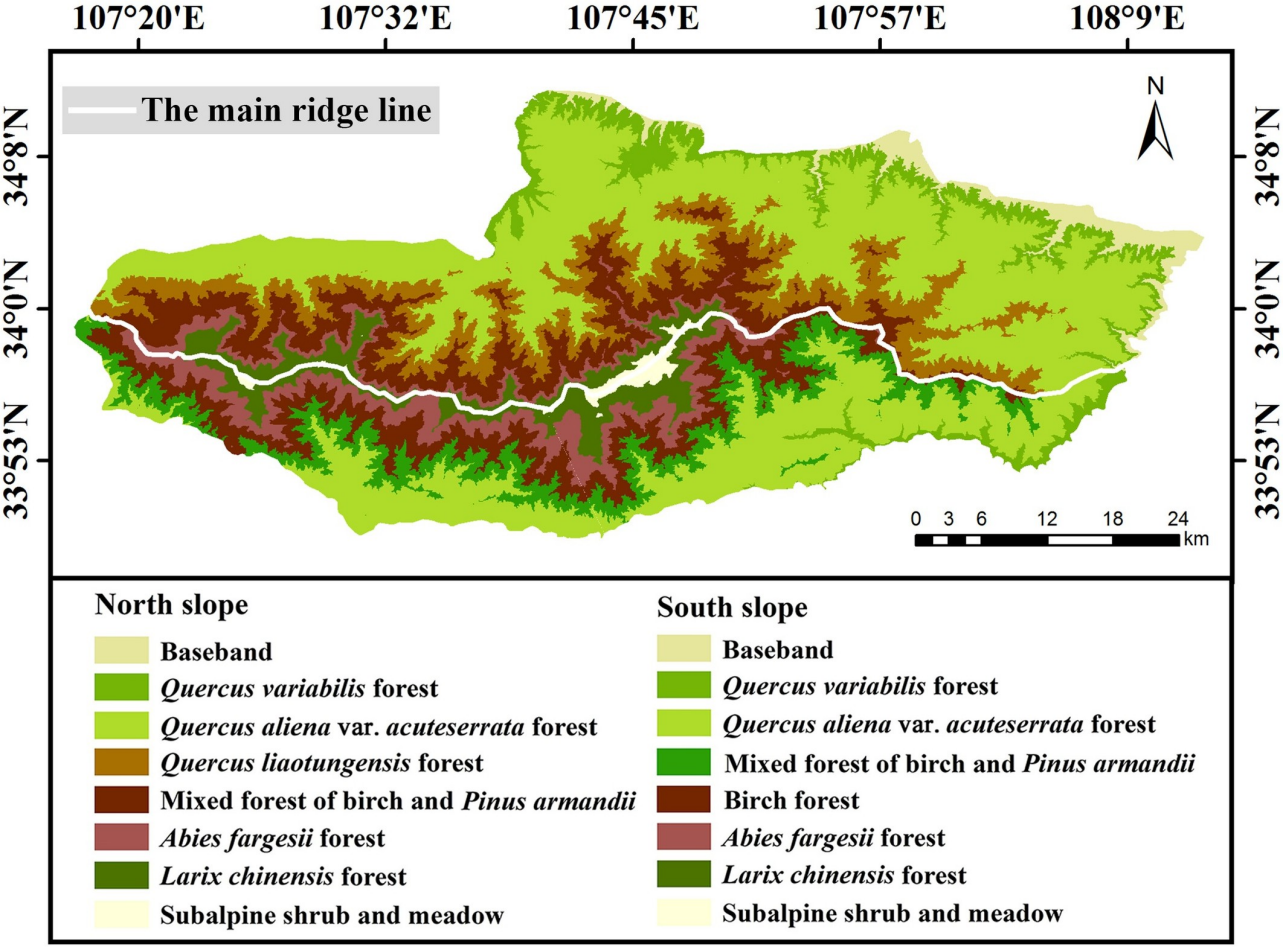
Terrain constraint factors with MAB distribution information on Taibai Mountain. The 1:10000 DSM data used to build the terrain constraint factor were generated from ZY-3 images.

In order to apply the prior knowledge provided by the MAB distribution information to select samples, terrain constraint factors were constructed by the following steps: 1) according to the 1:10,000 DSM data, the main ridgeline was extracted to divide the study area into the north and the south slopes; 2) the altitude distribution ranges of the vegetation formations were extracted according to the MAB; and 3) the terrain constraint factors were generated by superimposing factors, such as the altitude ranges of vegetation formations and DSM. The terrain constraint factors are shown in Fig 4.

**Fig 5:**
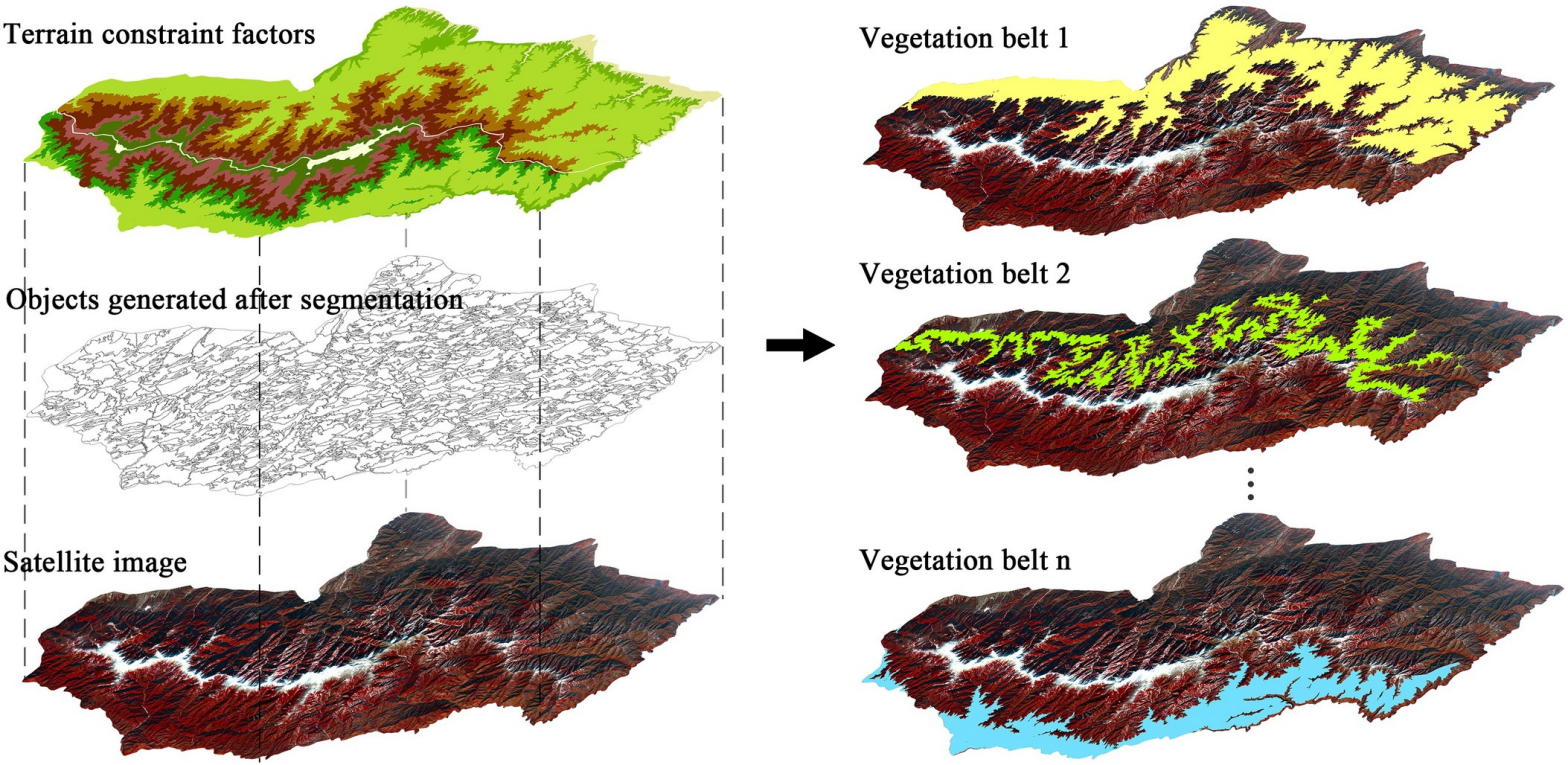
The process of generating vegetation belt layers with terrain constraint factors. The image was Landsat 8 image with a resolution of 15m, false color image (NIR, Red, Green), February 2017. The image is for illustrative purposes only.

By superimposing the terrain constraint factors and multi-scale segmentation layer on the image, the study area was divided into several vegetation belts for automatic sampling. The layering process is shown in Fig 5. Based on the idea of stratified sampling, the following sample selection was carried out.

#### (2) Automatic sample selection

**Fig 6:**
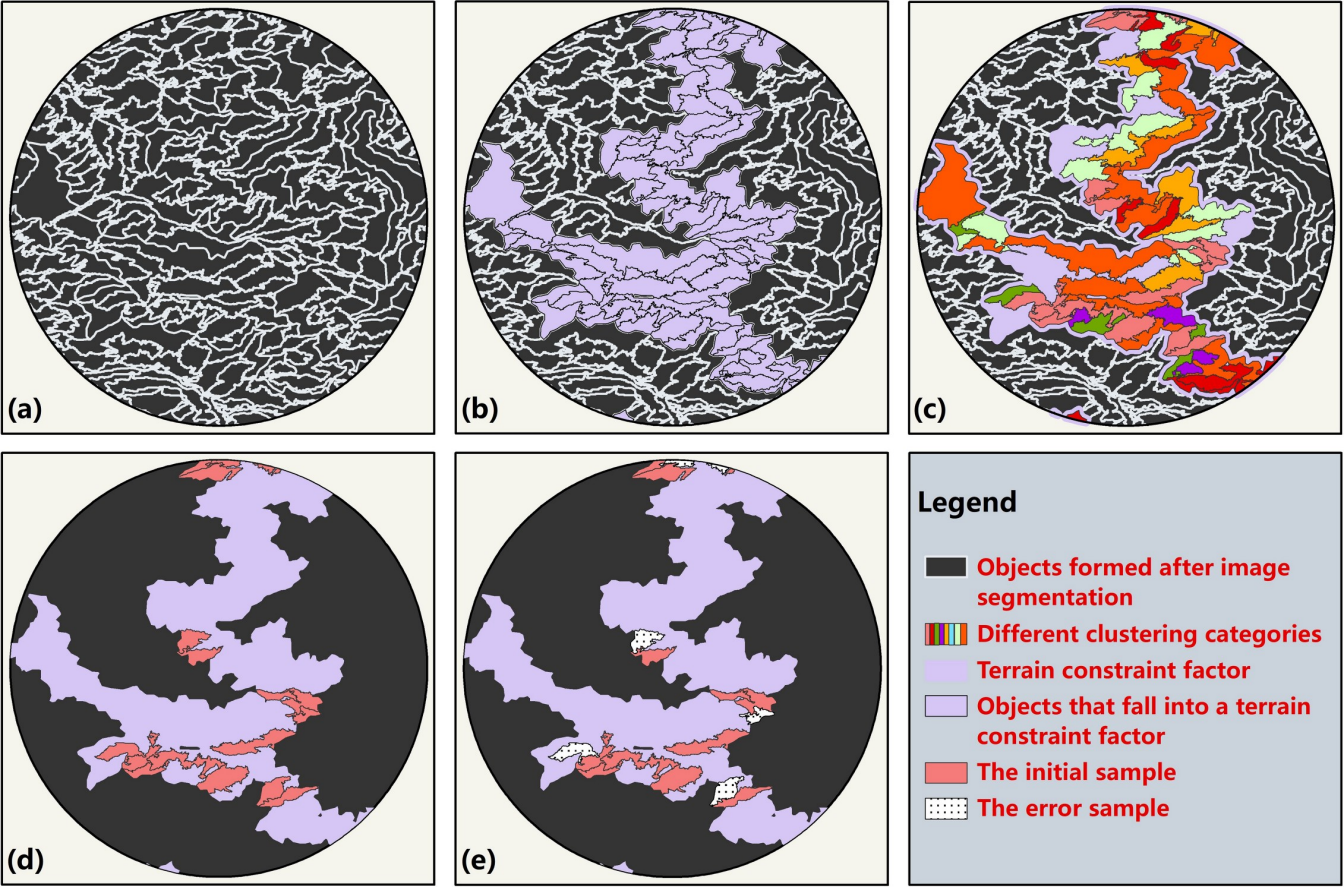
The process of automatic samples selection of a specific class. **(a)** Objects generated after the image multi-scale segmentation. **(b)** The *Pinus armandii* belt was superimposed on the segmentation results **(c)** Clustering of objects that fall within the belt **(d)** Initial samples (the most suitable category) selected from all the categories generated after clustering. **(e)** Verification and correction of initial samples.

The process of sampling is shown in Fig 6. Since the altitude ranges of vegetation on the north and south slopes were different, samples of the same vegetation formations were selected on the north and south slopes respectively.

The study area was divided into several vegetation belts by using the terrain constraint factors with MAB distribution information. Each belt was used to screen objects generated after the segmentation of scale 140 (Fig 6A). Taking the *Pinus armandii* belt at an altitude of 2000–2300 m on the south slope as an example, the belt was used to retain the objects falling within it as candidate objects of *Pinus armandii* (Fig 6B). These candidate objects contained characteristic information, including the area, ratio of length/width, mean (all bands), brightness, normalized difference vegetation index (NDVI) [54], difference vegetation index (DVI) [55], ratio vegetation index (RVI) [56], maximum difference measurement (Max. Diff), DSM, GLCM (Grey-Level Co-occurrence Matrix) contrast, and GLCM entropy. To prevent fragment polygons generated in the segmentation process from being selected into the sample database, the above candidate objects were filtered through the two indexes of area and length\width.

The initial samples of *Pinus armandii* were obtained by clustering the filtered candidate objects. In terms of selecting clustering methods, sample accuracy of the six commonly used machine learning clustering algorithms (SpectralClustering, Gaussian Mixture, AgglomerativeClustering, DBSCAN, Meanshift, KMeans) [57] was compared to obtain high-accuracy samples. The area containing all vegetation formations in Taibai Mountain was selected as the experimental area, the algorithm with the highest accuracy was selected for clustering. Moreover, in the clustering process: 1) the clustering number was three times the total number of possible vegetation formations near the altitude where the belt was located; 2) the characteristics of the objects used in clustering were the mean, brightness, NDVI, DVI, RVI, and Max. Diff.

**Fig 7:**
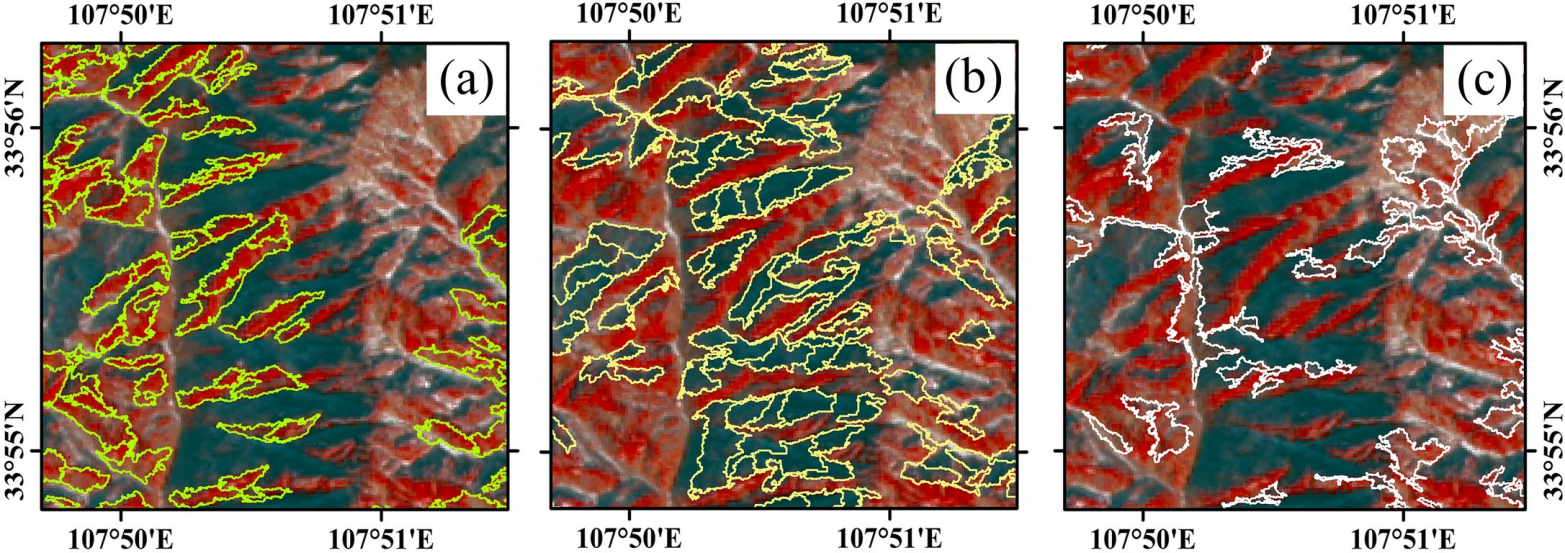
The top three categories for the number of objects in the clustering results of *Pinus armandii* belt. The image was Landsat 8 image with a resolution of 15m, false color image (NIR, Red, Green), February 2017. The image is for illustrative purposes only. The categories showed in **Column (a)**, **(b)** and **(c)** were the top three categories for the number of objects in the clustering result, and the categories in **Column (a)** were most suitable categories in the clustering results of *Pinus armandii* belt.

In the clustering results (Fig 6C), the top 3–5 categories of the number of objects were selected and compared with the image. The objects in the most suitable category were taken as samples of the current vegetation formation (*Pinus armandii*), as shown in Fig 7. And then, the samples were purified to eliminate outliers (Fig 6D). According to the Pauta Criterion [58,59], spectral values of the effective samples are primarily distributed around the mean values of the same class in the same region, and the occurrence of data outside the interval [μ_i_-3σ, μ_i_+3σ] is a small probability event. When the samples are within the interval, they are effective samples; otherwise, they are invalid samples. Based on the above principle, the objects were purified by Eq (1):

$$\|x_{i}-\mu_{i}\|\leq 3\sigma_{i},i=1,2,3,\ldots ,n$$ (1)

Where x_i_ is the brightness of the ith object, μ_*i*_ is the mean value of the class of the ith object, σ_*i*_ is the standard deviation.

The purified objects were used as undetermined samples of the vegetation formation (*Pinus armandii*). After collecting samples for all vegetation formations, the undetermined sample databases for north slope and south slope were established.

**Fig 8:**
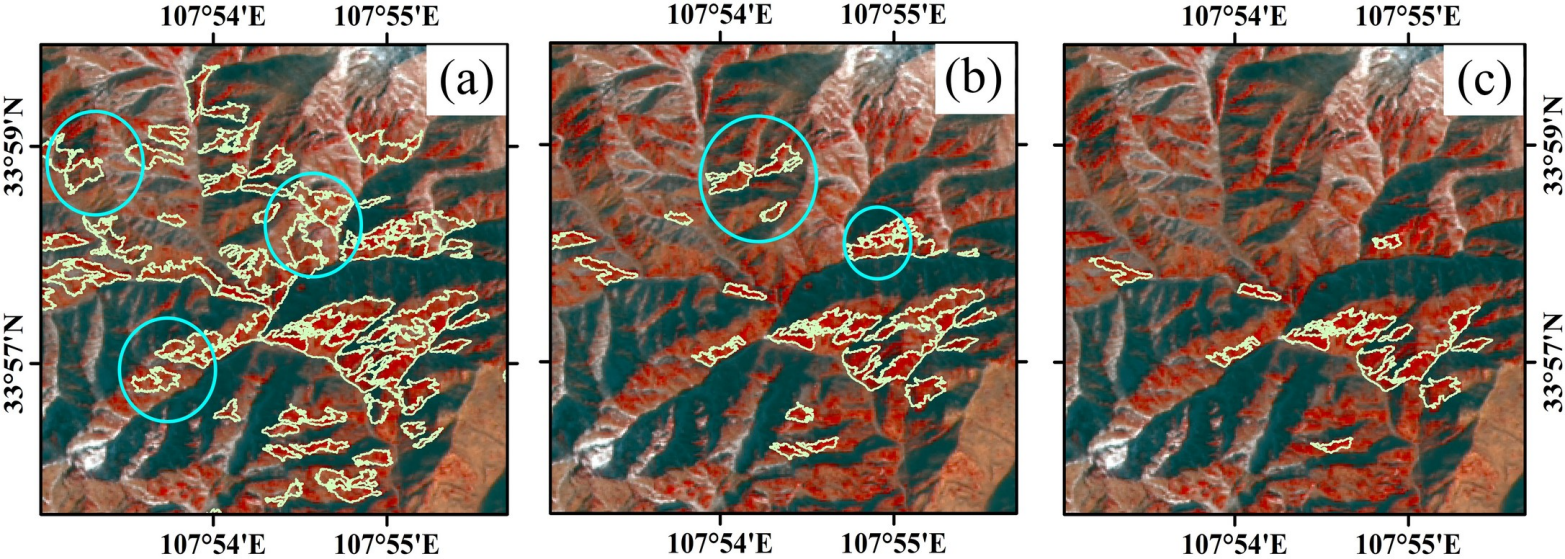
The correction process of *Pinus armandii* samples by iterative clustering. The image was Landsat 8 image with a resolution of 15 m, false color image (NIR, Red, Green), February 2017. **(a)** The first clustering result; **(b)** the second clustering result; **(c)** the last clustering result. The samples in the blue circles were the error samples. The image is for illustrative purposes only.

Due to the errors caused by clustering, it is necessary to verify and correct the undetermined samples (Figs 6E & 8). Therefore, we designed an iterative clustering method to correct the samples automatically. After obtaining the initial samples, we clustered the samples again and set the cluster number as 2. A more accurate category from the clustering results was selected as the samples for the first correction, and then clustered for the second time correction, and so on, until 1) the accuracy degree of the two categories in the results was roughly the same; 2) the number of samples was close to 120 (see S5 File for the code of sample selection process). The reasons for setting the number of samples of each vegetation formation as 120 were as follows: 1) references [60–63] show that the demand for the number of samples in object-oriented classification is less than that in pixel-based classification, which is 10–30 times of the number of image bands; 2) theoretically, the larger the number of samples, the higher the accuracy of classification. Since the number of bands of the images used in this paper is 4, we set the number of samples as 120 based on the above reasons.

### Object-oriented classification of vegetation

The classification process was also carried out according to the two layers of segmentation. Vegetation and non-vegetation were identified by the method of fuzzy classification [64] in Layer 1 in eCognition. The membership function constructed according to the same characteristics of buildings, roads, and other non-vegetation areas (low NDVI, high brightness values, distributed in low altitude areas) could completely extract non-vegetable objects. For example, when the NDVI*100 value of an object was less than -2, the membership value of this object for non-vegetation was 1; that is, this object must be non-vegetation. When the value was greater than 1, the membership value for non-vegetation was 0; when the value was in the (-2, 1) interval, the membership value was calculated according to the minor type membership function (as the value of X-axis gets closer to the right boundary, the membership value gets smaller; the shape is similar to but different from the Z-Shaped membership function) in the eCognition software. In addition, since the time phase of the image was in winter, the variation range of the NDVI value was small and relatively concentrated. After repeated comparisons, the interval (-2, 1) was selected. To prevent shadows or alpine snow from being misclassified as non-vegetation objects, not only NDVI but also brightness and DSM should be considered when classifying.

In Layer 2, the “class-related-features” function in eCognition was used to transfer classification information between layers firstly, so that all objects in Layer 2 could inherit the vegetation and non-vegetation attributes of Layer 1. Then, RF classifier and KNN classifier were used to classify objects whose attributes were vegetation. In the classification process using RF, features were first sorted in order of importance (with the default RF parameters) using the “feature_importance” function (an output variable of RF algorithm) [65], and the top eight features were utilized in the classification (accounting for 95.2%, see S4 Fig for the ranking of the features), including the mean of the band (NIR, Red), vegetation index (NDVI, DVI, RVI), Max. Diff, texture feature (contrast), and brightness. Then the classifier parameters were adjusted by calculating F1-score [66]. The F1-score is a measure of accuracy, and it is the harmonic mean of the precision and recall, where an F1 score reaches its best value at 1. The final parameter combination adopted was {Max_depth = 19, Max_feature = 8, N_estimators = 20, Min_samples_leaf = 2}, which was the parameter combination corresponding to the maximum value of calculated F1-score. In the classification process using KNN, the same features were used as above, and the adopted parameter combination after calculating F1-score was {n_neighbors = 9, weights = ‘uniform’, algorithm = ‘auto’, p = 2, metric = ‘minkowski’}. During the classification process, 120 samples of each vegetation formation were randomly selected from the corrected sample database.

### Accuracy verification of samples and classification results

To comprehensively explain the quality of the selected samples, sample accuracy was analyzed from three perspectives: north\south slopes of Taibai Mountain, west\middle\east regions of Taibai Mountain, and overall accuracy of the entire Taibai Mountain. The accuracy of the selected samples was calculated by the ratio correct number/total number of samples and verified by combining with the field sampling data.

The accuracy of the classification results was verified by comparing the classification results of 1000 points (randomly generated in the research area) with the visual interpretation results (points where the classification results did not match the visual interpretation results would be marked as error points), and calculating overall accuracy, user accuracy, producer accuracy, and kappa coefficient [67]. Overall accuracy is the probability that an individual will be correctly classified by a test, and it is computed by dividing the total number of correctly classified objects by the total number of reference objects. Producer accuracy computed by dividing the number of correctly classified objects in each category by the number of reference objects known to be of that category; this value represents how well reference objects of the vegetation formation are classified. User accuracy is computed by dividing the number of correctly classified objects in each category by the total number of objects classified in that category; this value represents the probability that an object classified into a given category actually represents that category on the ground. The Kappa coefficient is used to measure the agreement between two sets of categorizations of a dataset while correcting for chance agreements between the categories; it can range from -1 to 1, and the closer it gets to 1, the better the result of the classification.

## Results

### Accuracy of different clustering methods for sampling

Table 3 shows the comparison of sample accuracy obtained by the first clustering of each clustering algorithm. From the perspective of mean value, maximum value (the underlined value in Table 3) and minimum value to analyze the performance: among the mean value of sample accuracy obtained by each algorithm, the value of KMeans was largest; and the maximum values of sample accuracy appeared most frequently in KMeans; furthermore, the minimum value of sample accuracy did not appear in KMeans. It can be seen from the above analysis that KMeans had the best performance, so the KMeans algorithm was used to select samples.

**Table 3:** Comparison of the sample accuracies of the six clustering algorithm.

Accuracy	Spectral Clustering	Gaussian Mixture	Agglomerative Clustering	DBSCAN	MeanShift	KMeans
*Quercus variabilis*	0.81	0.85	0.79	0.83	0.77	0.82
*Quercus aliena* var.*acuteserrata*	0.86	0.93	0.89	0.84	0.76	0.86
*Quercus liaotungensis*	0.76	0.84	0.72	0.78	0.74	0.84
*Pinus armandii*	0.76	0.81	0.81	0.70	0.78	0.94
Birch forest	0.90	0.79	0.86	0.74	0.50	0.79
Mixed forest	0.79	0.87	0.71	0.77	0.73	0.84
*Abies fargesii*	0.86	0.89	0.88	0.67	0.79	0.96
*Larix chinensis*	0.83	0.78	0.82	0.68	0.81	0.88
Subalpine shrub and meadow	0.89	0.83	0.81	0.61	0.76	0.89
**Mean**	0.83	0.84	0.81	0.74	0.74	0.87

(Underlined values are the maximum accuracies of samples obtained by the six clustering methods per vegetation class)

### Accuracy of the sample database

**Fig 9:**
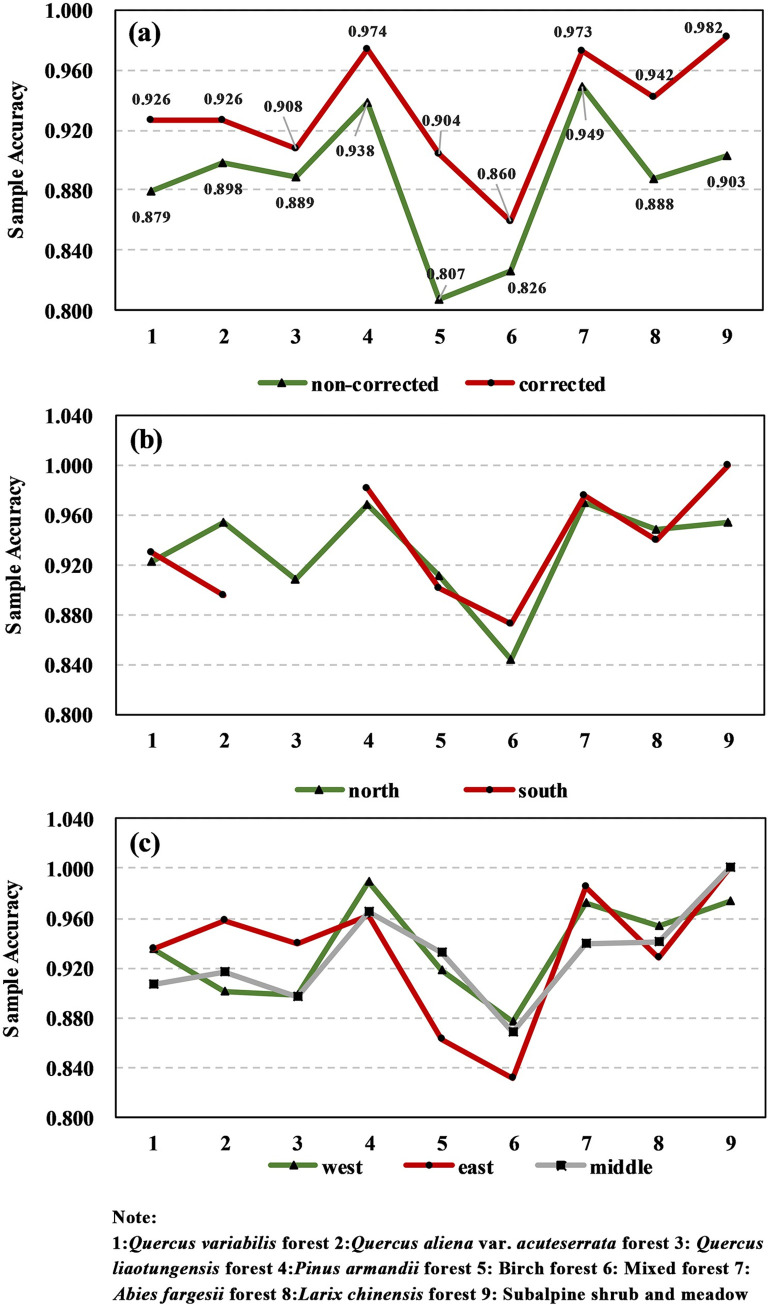
Sample accuracies in different perspectives of Taibai Mountain. **(a)** the comparison of sample accuracy before and after correction of the entire Taibai Mountain; **(b)** the sample accuracy on the north\south slope of Taibai Mountain; **(c)** the sample accuracy in the west \middle\east region of Taibai Mountain.

Fig 9A shows the comparison of the sample accuracy before and after the iterative correction of the entire Taibai Mountain. As can be seen from the figure, the sample accuracy of each vegetation formation was significantly improved after correction, indicating that the iterative clustering method can effectively correct samples. Among the sample accuracy before and after correction of each vegetation formation, the accuracy of the birch forest, *Larix chinensis*, and *subalpine shrub and meadow* increased the most. Except for coniferous broad-leaved mixed forests, the accuracy of corrected samples of all vegetation formations was above 0.900. The average accuracy of corrected samples was 0.933, which was significantly higher than that of uncorrected samples (0.886). The corrected sample accuracy of the coniferous forest (0.963) was higher than that of the broad-leaved forest (0.916).

Fig 9B shows the corrected sample accuracy of the north and south slope of Taibai Mountain. The average sample accuracy of the north slope was 0.931, and that of the south slope was 0.937. The accuracy of *Quercus aliena* var.*acuteserrata* varied greatly between the north and south slope, and the value of the north slope (0.954) was higher than that of the south slope (0.895). The accuracy of subalpine shrub and meadow was also significantly different, the value of the south slope (1.000) was higher than that of the north slope (0.953). Among all the sample accuracies, the minimum value was for the coniferous and broad-leaved mixed forest on the north slope (0.844), and the maximum value was for the meadow shrub on the south slope (1.000). In addition, there was no sample data for the *Quercus liaotungensis* forest on the south slope because it only grows on the north slope of Taibai Mountain.

Fig 9C shows the corrected sample accuracy of the west\middle\east regions of Taibai Mountain. The rule for the three regions was roughly similar, which was the accuracy of broad-leaved forests was lower than that of coniferous forests. However, there were also some differences. In the west region, the accuracy of *Pinus armandii* (0.989) was higher than the average accuracy of the other two regions (0.964), but the accuracy of shrub meadow (0.974) was lower than the average accuracy of the other two regions (1.000). In the east region, the accuracies of *Quercus aliena* var.*acuteserrata* (0.958) and *Quercus liaotungensis* (0.939) were higher than the average accuracies (0.909, 0.898) in the other two regions, but the accuracies of birch forest (0.863) and mixed forest (0.832) were lower than the average accuracies (0.925, 0.873) in the other two regions. In the middle region, the accuracies of *Quercus variabilis* (0.907) and *Abies fargesii* (0.940) were lower than the average accuracies (0.935, 0.979) in the other two regions. Among all the sample accuracies, the minimum value was for the coniferous and broad-leaved mixed forest in the east region (0.832), and the maximum value was for the shrub meadow in the east and middle region (1.000). Overall, the sample accuracy of the broad-leaved forest was lower than that of the coniferous forest, but there were significant differences in different distribution regions.

### Classification results of corrected samples

**Fig 10:**
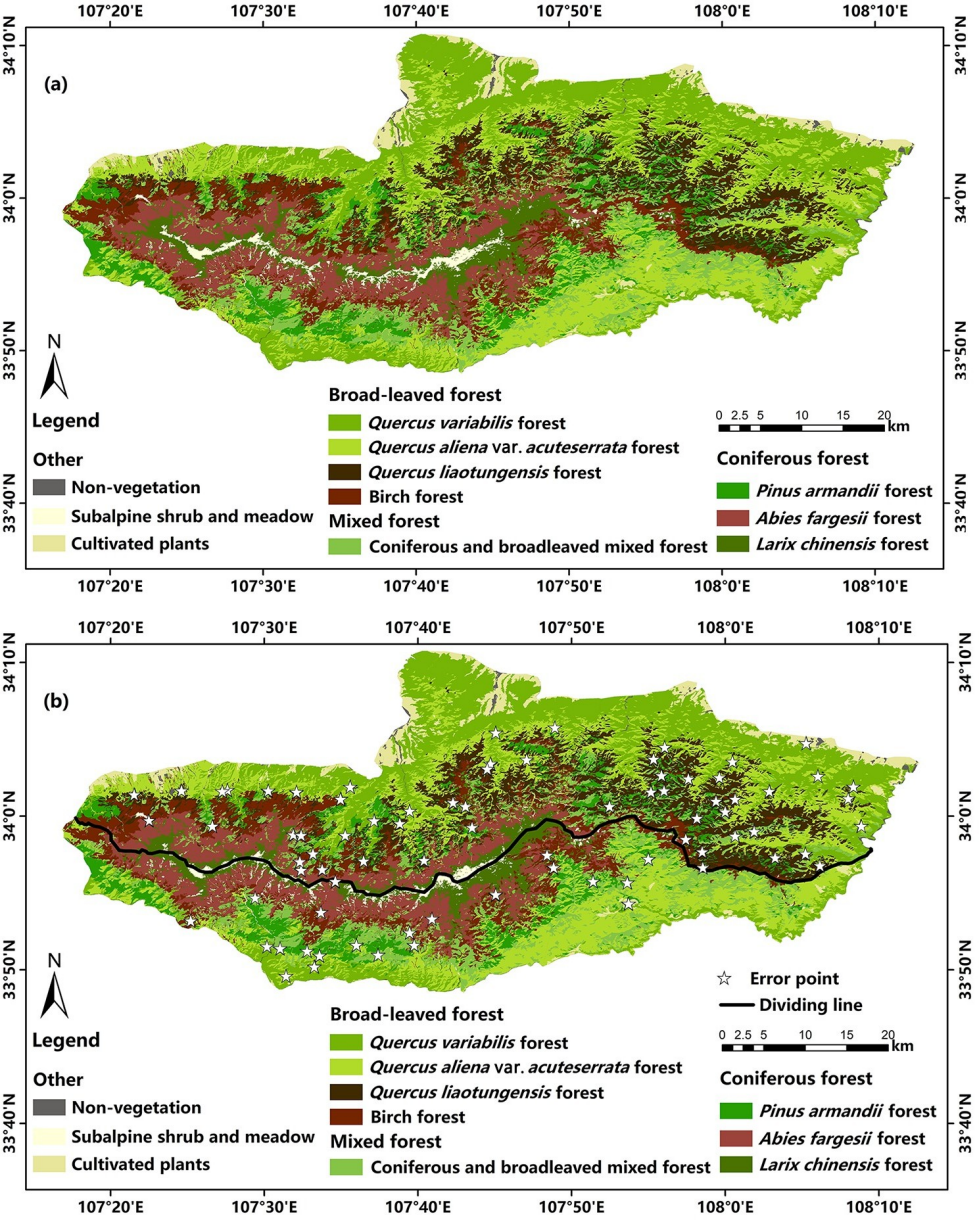
Classification results of the corrected samples using RF classifier. **(a)** Vegetation classification results, **(b)** distribution of error points (white stars).

From the confusion matrix shown in Tables 4 and 5, it can be seen that the overall classification accuracy of RF was 92.2% and the kappa coefficient was 0.910. The overall classification accuracy of KNN was 87.4%, and the kappa coefficient was 0.855. The above means that the classification result of RF classifier was better than that of KNN classifier. The classification result of RF is shown in Fig 10A, the distribution of error points is shown in Fig 10B. There were 79 error points in 1000 randomly generated points. According to the distribution of error points, it can be seen that: 1) the number of error points on the north slope was higher than that on the south slope, 2) the number of error points in the eastern region was higher than that in the western and middle regions, and 3) the numbers of error points were higher in the mixed forest and broad-leaved forest. As shown in Table 4, the user accuracy of the coniferous forest was higher than that of the broad-leaved forest. This rule was consistent with our analysis of sample accuracy, which also indicated that the classification accuracy depended on the sample accuracy to a large extent.

**Table 4:** Confusion matrix of the classification results of the RF classifier.

Class	*Quercus variabilis*	*Quercus aliena* var. *acuteserrata*	*Quercus liaotungensis*	*Pinus armandii*	Birch forest	Mixed forest	*Abies fargesii*	*Larix chinensis*	Subalpine shrub and meadow	Cultivated plants	Non-vegetation	Total	User accuracy
*Quercus variabilis*	197	11	1	0	4	7	0	0	0	0	0	220	89.5%
*Quercus aliena* var.*acuteserrata*	4	165	3	0	2	4	0	0	0	1	0	179	92.2%
*Quercus liaotungensis*	0	7	87	1	1	4	0	0	0	0	0	100	87.0%
*Pinus armandii*	0	0	0	64	0	2	0	0	0	0	0	66	97.0%
Birch forest	0	2	0	0	86	7	1	0	0	0	0	96	89.6%
Mixed forest	0	1	0	3	2	109	2	0	0	1	0	118	92.4%
*Abies fargesii*	0	0	0	0	0	4	120	0	0	0	0	124	96.8%
*Larix chinensis*	0	0	0	0	0	1	0	46	2	0	0	49	93.9%
Subalpine shrub and meadow	0	0	0	0	0	0	0	0	18	0	0	18	100.0%
Cultivated plants	0	0	0	0	0	0	0	0	0	26	0	26	100.0%
Non-vegetation	0	0	0	0	0	0	0	0	0	0	4	4	100.0%
Total	201	186	91	68	95	138	123	46	20	28	4	1000	
Producer accuracy	98.0%	88.7%	95.6%	94.1%	90.5%	79.0%	97.6%	100.0%	90.0%	92.9%	100%		

Total accuracy: 92.2%; coefficient of kappa: 0.910.

**Table 5:** Confusion matrix of the classification results of the KNN classifier.

Class	*Quercus variabilis*	*Quercus aliena* var. *acuteserrata*	*Quercus liaotungensis*	*Pinus armandii*	Birch forest	Mixed forest	*Abies fargesii*	*Larix chinensis*	Subalpine shrub and meadow	Cultivated plants	Non-vegetation	Total	User accuracy
*Quercus variabilis*	166	23	5	1	0	0	0	0	0	2	0	197	84.3%
*Quercus aliena* var.*acuteserrata*	11	146	2	0	1	6	0	0	0	1	0	167	87.4%
*Quercus liaotungensis*	1	16	89	3	4	6	0	0	0	0	0	119	74.8%
*Pinus armandii*	0	4	1	116	2	0	1	0	0	0	0	124	93.5%
Birch forest	0	0	3	1	98	4	8	2	0	0	0	116	84.5%
Mixed forest	0	6	1	1	0	84	0	1	0	0	0	93	90.3%
*Abies fargesii*	0	0	0	0	3	1	81	0	0	0	0	85	95.3%
*Larix chinensis*	0	0	0	0	0	0	1	55	3	0	0	59	93.2%
Subalpine shrub and meadow	0	0	0	0	0	0	0	1	9	0	0	10	90.0%
Cultivated plants	0	0	0	0	0	0	0	0	0	27	0	27	100%
Non-vegetation	0	0	0	0	0	0	0	0	0	0	3	3	100%
Total	178	195	101	122	108	101	91	59	12	30	3	1000	
Producer accuracy	93.3%	74.9%	88.1%	95.1%	90.7%	83.2%	89.0%	93.2%	75%	90.0%	100%		

Total accuracy: 87.4%; coefficient of kappa: 0.855

Moreover, according to the MAB distribution information and the classification results presented in this paper, the distribution characteristics of vegetation were approximate as follows: 1) the *Quercus liaotungensis* forest only grew on the north slope of Taibai Mountain, and the number gradually increased from west to east. 2) The number of birch forests gradually decreased from west to east, and birch forests were always mixed with the *Pinus armandii* forest and *Abies fargesii* forest. In addition, due to more dead branches and weak growth inside the *Betula albosinensis* var. *septentrionalis* forest, it may eventually be replaced by the *Abies fargesii* forest. Therefore, this paper did not further subdivide *Betula albosinensis* and *Betula albosinensis* var. *septentrionalis*, but unified them as birch forest. 3) The basal belt of the north slope was broader than that of the south slope, so the area of cultivated plants on the north slope was more extensive than that on the south slope. And 4) the eastern part of Taibai Mountain was gentler than the western and middle regions and had relatively few vegetation formations. The area of subalpine shrub meadow and *Abies fargesii* forest in the eastern region was much smaller than that in the western and middle regions. By consulting the relevant literature, the above distribution rules were consistent with the results of previous studies [68–75].

## Discussion

In the process of vegetation mapping, many studies have realized automatic improvement from manual visual interpretation to computer image classification. However, this process still involves human-computer interaction, especially the selection of samples. As algorithms such as deep learning are proposed, many models are moving from manual intervention to full-step automation, but still cannot meet the requirements of fine-scale for vegetation mapping. For example, Shorter *et al*. [23] proposed a method using a novel color quantization technique coupled with color invariant scheme to identify vegetation. By analyzing the spectral characteristics of vegetation and using the difference between NDVI and background values to set thresholds, Yao *et al*. [24] proposed the Hyperplanes for Plant Extraction Methodology to achieve automatic extraction of vegetation. The minimum unit of the above research classification system is usually vegetation type group or vegetation type, which is of relatively weak value compared with more detailed studies [76–78]. Previous studies have shown the contradiction between fineness and automation in the classification process. Although the method using prior maps can alleviate this problem to a large extent, it is not applicable to un-interpreted regions, and there is also the problem of error propagation. Actually, compared with other land features, mountain vegetation is more challenging to extract from remote sensing images, especially when classification systems are specific to vegetation formations, for the following reasons: 1) the boundaries of different vegetation formations are not generalized and sometimes fuzzy; 2) A given vegetation formation may have different phenology due to vegetation seasonal or composite classes; 3) shadow effect from nearby trees or mountains [4,79–81]. Therefore, under the above background, this paper sought a method that can not only reduce manual intervention but also guarantee the classification accuracy.

In the process of automating the classification, there are many constraints, including the collection of prior knowledge and data, the selection of samples, the selection of classifiers and the adjustment of parameters, etc. This study mainly focused on solving the problem of automatic selection of samples. The main innovation is that compared with traditional manual sample selection, an automatic sample selection method was proposed in the context of fine-scale vegetation mapping. Therefore, the "high automation" in the manuscript mainly refers to the sample selection process, and human intervention still existed in the segmentation and classification process. For the segmentation process, although there are plug-ins for automatic parameter adjustment in eCognition, the accuracy is usually guaranteed in flat areas [82,83]. In order to ensure accuracy, we manually set the segmentation parameters and band weight. For the classification process, the classification of Layer 1 was completed by the fuzzy membership function in eCognition. The manual operation is to select the function type (such as triangular, trapezoidal, Gaussian, generalized bell, etc.) and set the endpoint values. The classification of Layer 2 was completed by using RF classifier. As mentioned in the method section, features used were selected by feature sorting, and the optimal parameter combination was selected by calculating F1-score. The process of using the RF classifier was almost automatic.

While realizing the automatic classification of vegetation, it is also very important to ensure the overall high accuracy. From the analysis of the sample accuracy, it can be seen that the iterative clustering can effectively correct the sample, but there was still noise in the result. As can be seen from Fig 9, the sample accuracies of different vegetation formations varied with different perspectives, which may be caused by the following reasons:

1) The interiors of land cover areas and larger patches are generally more ecologically stable. In the eastern part of Taibai Mountain, the distribution ranges of broad-leaved forests were larger than those in the middle and western regions, so the sample accuracy was relatively high.

2) In the eastern region, the altitude difference is smaller than that in the middle and western regions, and areas above 2300m account for only 4% of the total area; so the distribution ranges of pure birch forest and coniferous forest are relatively small, which means that most coniferous forests and birch forests in the eastern high-altitude region are formed as mixed forests. So the accuracy of birch forest and mixed forest was lower than the average accuracy in the west and middle regions.

Inappropriate samples are identified as the main source of errors in many classification processes [84]. However, completely accurate samples are not easy to obtain because they require a lot of labor or time, so it is necessary to reduce the impact of incorrect samples effectively [85,86]. Previous studies have shown that when using RF and SVM (Support Vector Machine) classifier to classify samples with noise less than 25%-30%, sample noise has little impact on the classification results [87,88]. The results from Tables 4 and 5 showed that the RF classifier was more robust to outliers than the KNN classifier. The RF classifier was selected to realize vegetation classification from the perspective of classification performance. Although RF classifier could effectively reduce the impact of sample noise on classification, there were still some misclassification cases, as shown in Table 4, which were mainly concentrated in the mixed forest and broad-leaved forest. The reasons may be as follows:

1) There are two kinds of objects formed after image segmentation: pure objects composed of pure pixels and non-pure objects composed of mixed pixels, among them, non-pure objects are more likely to be misclassified; the objects of mixed forests were composed of a mixture of pixels whose properties were coniferous and broad-leaved forests, so they were more easily misclassified.

2) Ecologically, the junctions of different land cover classes are fragile areas. The coniferous and broad-leaved mixed forest was located at the junction of the coniferous forest belt and the broad-leaved forest belt, and the definition was relatively fuzzy.

3) Because the time phase of the image selected for classification was winter, the image features of various vegetation formations in the broad-leaved forest were similar, and misclassification was likely to occur near the boundaries of the altitudinal belts.

(4) Compared with broad-leaved forests, coniferous forests had brighter colors and more obvious textures in the images, so misclassification occurred less in coniferous forests, which means the classification accuracy of coniferous forest was higher than that of broad-leaved forest.

Previously, we studied whether assisting MAB distribution information in manual sample selection can improve classification accuracy [52]. The result showed that the overall classification accuracy of the samples selected manually assisted with MAB distribution information was 92.9%, which was 10% higher than that of the samples selected without MAB. It can be seen that stratified sampling assisted by MAB distribution information can effectively improve the accuracy. In combination with the previous studies [52], the method in this paper has gradually improved the efficiency of sample selection, from manual sample selection to manual sample selection based on MAB distribution information to highly automated sample selection based on MAB distribution information. More importantly, the method in this paper not only improved the efficiency but also guaranteed the classification accuracy. Compared with the visual interpretation or manual sample selection, this method did not require a lot of interpretation experience or time. We can complete a series of sample selection and classification with high automation assisted by MAB distribution information, which are easy to obtain. In other words, this method has certain versatility for mountain areas with obvious vertical distribution rules of vegetation.

However, there are still some problems affecting the accuracy, such as the following two points: 1) the sample accuracy of mixed forests (especially in the eastern region) was the main reason for lowering the average sample accuracy; 2) the terrain constraint factors that played a key role in the sample selection only considered the altitude factor in this study, which may be able to further improve the sample accuracy by adding other terrain factors such as slope and slope direction. How to solve these problems will be the focus of our next research.

## Conclusions

The main purpose of this research is to alleviate the contradiction between automation and fitness in remote sensing vegetation mapping. Based on high-resolution remote sensing images, we used MAB distribution information as prior knowledge to construct terrain constraint factors for stratification and achieved sample selection with high automation based on the idea of stratified sampling. For the noise generated in the process of sampling, an iterative clustering method was designed to correct the noise automatically. The average sample accuracy after the correction was 0.933, which was significantly improved compared with the average sample accuracy before the correction of 0.886. The overall accuracy of classification with the RF classifier was 92.2%, and the kappa coefficient was 0.910. The main finding is that the method used in this paper can automatically select samples and realize fine-scale vegetation classification with high accuracy, and has universal applicability for mountain areas with obvious vertical distribution rules of vegetation. In conclusion, this method can be applied to fine-scale vegetation mapping in large areas with high accuracy and efficiency.

## Supporting information

S1 Fig A broken line graph of the mean-variance and segmentation scale. (TIF)

S2 Fig The segmentation results when the segmentation scale is 360 (a) and 140 (b). The image was Landsat 8 image with a resolution of 15m, false color image (NIR, Red, Green), February 2017. The image is for illustrative purposes only.
(TIF)

S3 Fig Segmentation results under different band weight combinations (NIR, Red, Green, Blue, DSM, texture). The image was Landsat 8 image with a resolution of 15m, false color image (NIR, Red, Green), February 2017. The image is for illustrative purposes only.
(TIF)

S4 Fig The ranking of feature importance using RF classifier. (TIF)

S1 File The Python3 code for the automatic sample selection process. (TXT)
