# Supplementary figures and images for: Automatic classification of fine-scale mountain vegetation based on mountain altitudinal belt
Source: PLoS One. 2020 Aug 25;15(8):e0238165. doi: 10.1371/journal.pone.0238165 (PMC7447069; doi:10.1371/journal.pone.0238165)

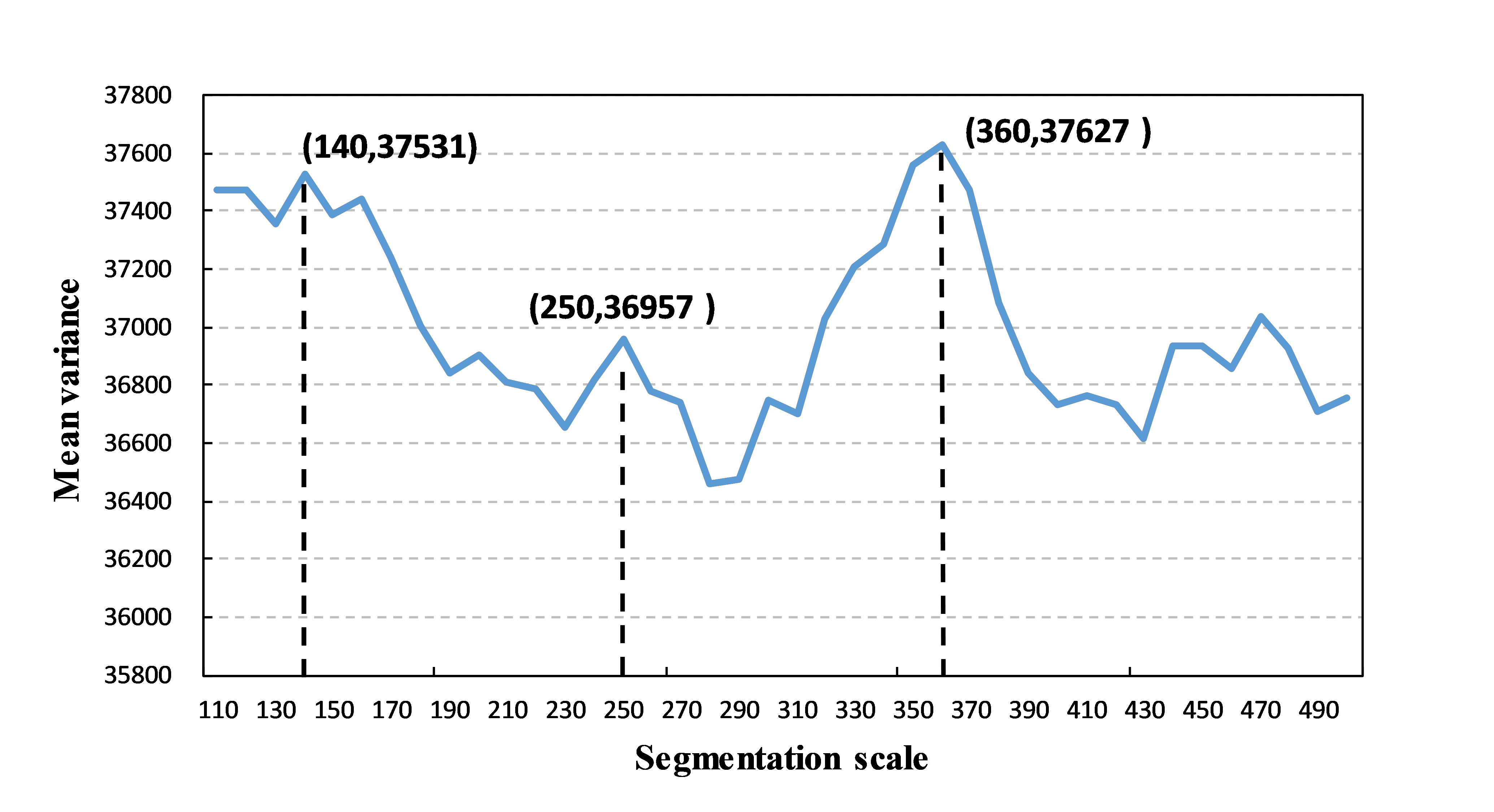

Supplement: S1 Fig — (TIF) [file pone.0238165.s001.tif]

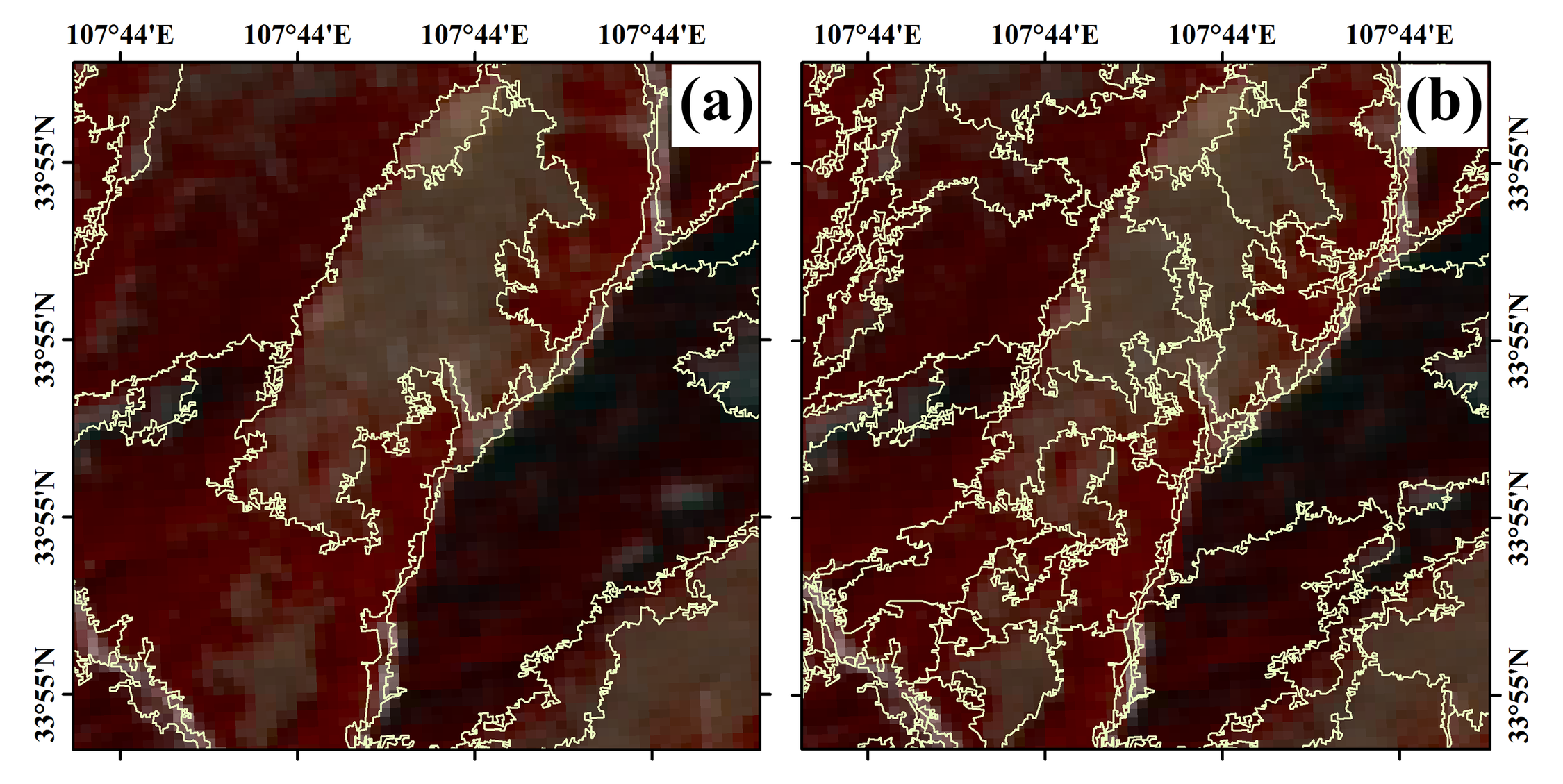

Supplement: S2 Fig — The segmentation results when the segmentation scale is 360 (a) and 140 (b). The image was Landsat 8 image with a resolution of 15m, false color image (NIR, Red, Green), February 2017. The image is for illustrative purposes only. (TIF) [file pone.0238165.s002.tif]

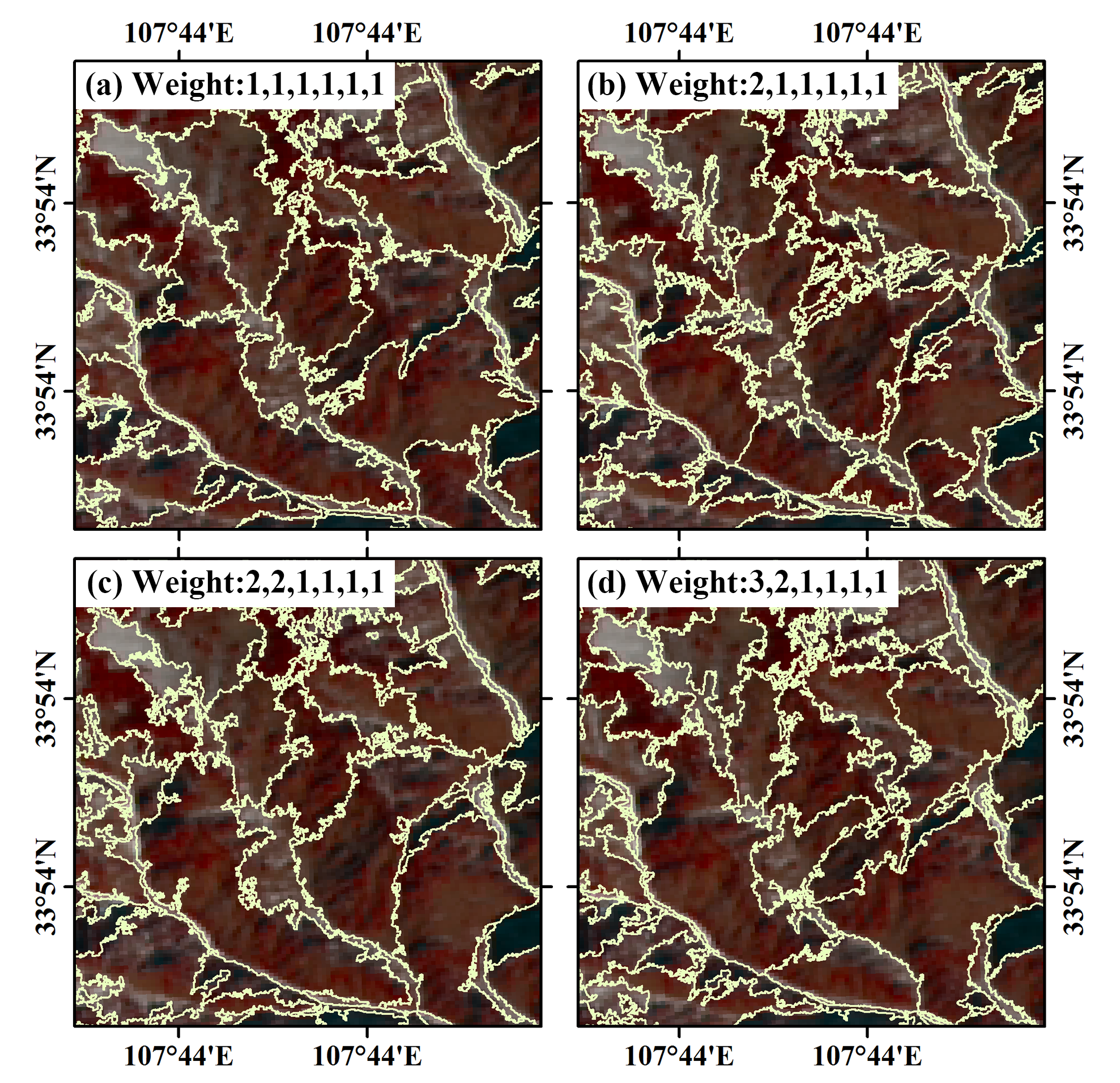

Supplement: S3 Fig — The image was Landsat 8 image with a resolution of 15m, false color image (NIR, Red, Green), February 2017. The image is for illustrative purposes only. (TIF) [file pone.0238165.s003.tif]

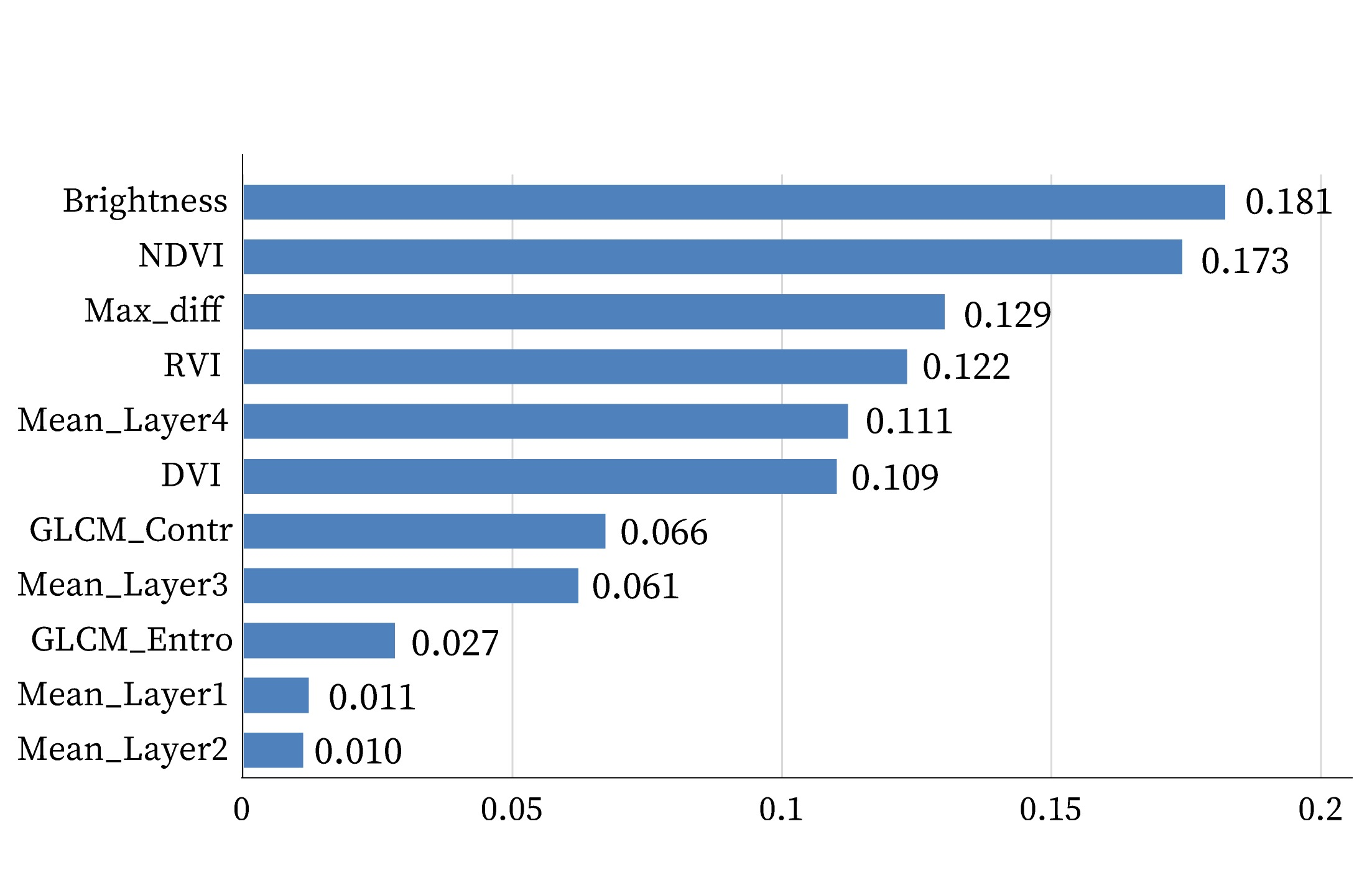

Supplement: S4 Fig — (TIF) [file pone.0238165.s004.tif]
